# Synthesis and Decarboxylation of Functionalized 2-Pyridone-3-carboxylic Acids and Evaluation of their Antimicrobial Activity and Molecular Docking

**DOI:** 10.22037/ijpr.2021.114749.15018

**Published:** 2021

**Authors:** Elmira Meghrazi Ahadi, Homa Azizian, Vaezeh Fathi Vavsari, Atousa Aliahmadi, Zeinab Shahsavari, Hamid R. Bijanzadeh, Saeed Balalaie

**Affiliations:** a *Peptide Chemistry Research Institute, K. N. Toosi University of Technology, Tehran, Iran. *; b *Department of Medicinal Chemistry, School of Pharmacy, International Campus, Iran University of Medical Sciences, Tehran, Iran. *; c *Medicinal Plant and Drug Research Institute, Shahid Beheshti University, Tehran, Iran. *; d *Department of Environmental Sciences, Faculty of Natural Resources and Marine Sciences, Tarbiat Modares University, Tehran, Iran. *; e *Medical Biology Research Center, Kermanshah University of Medical Sciences, Kermanshah, Iran.*

**Keywords:** Multicomponent reactions; Antibiotics, 2-Pyridone, Decarboxylation reaction, Induced fit docking, ADME properties, Molecular dynamic

## Abstract

The functionalized 2-pyridone-3-carboxylic acids were synthesized starting from 3-formylchromone. Meanwhile, a decarboxylation reaction of 2-pyridone-3-carboxylic acid was performed by potassium carbonate in toluene. All compounds were evaluated against two Gram-negative bacteria (*Escherichia coli (E. coli)*,* Acinetobacter baumannii (A. baumannii*)) and two Gram-positive (*Staphylococcus aureus (S. aureus)*) and fungus (*Candida albicans* (*C. albicans)*) using serial broth dilution method. The antimicrobial screening revealed that *S. aureus* is the highest sensitive microorganism towards the synthesized compounds. Among all analogs, derivatives,** 4p **and** 5c** showed excellent activities in comparison with the other compounds against *S. aureus*. Molecular docking showed that the most active anti *S. aureus* are compounds **4p** and **5c** exhibiting primary interaction as with fluoroquinolones by cross-linking over DNA gyrase active site via metal ion bridge and H-bonding interaction with Ser84 and Glu88 from GyrA subunit along with Arg458 and Asp437 located at GyrB subunit. In addition, based on the molecular dynamic simulation as like the standard fluoroquinolones, the mentioned compounds were stabilized for significant amount of simulation time over DNA gyrase which potentiate the importance of the mentioned residues in the DNA gate region of DNA gyrase.

## Introduction

Designing new antibacterial agents, especially the selective ones, has become urgent, important and challenging research because of the antibiotic resistance, the appearance of resistant bacteria, and the increase of death as a result of infections (1).

The 2-pyridone motif is the core of many vital bioactive compounds and medicines, such as Pirfenidone and Flourofenidone, and easy preparation of pyridines functionalized at specific positions is important to synthetic and medicinal chemistry (2, 3). 2-Pyridones are mainly inspiring substrates for the synthesis of antimicrobial (4), antifungal (5), antiviral (6), anti-Alzheimer (7), anti-inflammatory (8), and cardiotonic (9) agents. Several remarkable antimicrobial agents consist of the 2-pyridone ring have been reported such as ABT-719, A-78830 (10-12), curlicide FN075 (13, 14), and nybomycin (15). Moreover, the 2-salicyloyl scaffold is the common potent moiety in the structure of many pharmacological and biologically active natural products such as flavonoid (16), isopestacin (17), pyrrolomycin C (18), and paenol (19) having potent antimicrobial activity.

**Figure 1:**
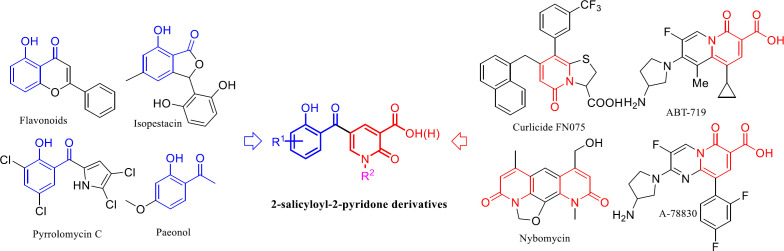
The new molecular hybridization of 2-salicyloyl and 2-pyridones

These encouraging results led us to design a new scaffold consist of both 2-salicyloyl and 2-pyridones moiety through molecular hybridization strategy to develop a new antibacterial agent (Figure 1). 

In this way, designing new structures comprising 2-pyridone is attractive research since by introducing substituents on different positions of its structure, the bioactivity may be changed. Moreover, substituted 2-pyridones have been employed as synthons for the preparation of numerous valuable N-rich heterocycles, including azachromones (20), quinoline, quinolone (21) and so on.

Various methodologies have been published for the preparation of 2-pyridones (22-24). Based on our experience, designing innovative synthetic routes to introduce carboxylic acid, hydroxybenzoyl, and *N*-aryl substituents in the 2-pyridone motif is highly preferred. Such ideal synthesis is achievable through the three-component reaction between 3-formylchromone, primary amines, and Meldrum’s acid in our laboratory (25). Due to its productivity in the preparation of new organic derivatives, decarboxylation reaction has been considered extensively among chemists. In many cases, by introducing substituents on rings, decarboxylation is accomplished to achieve the desired products. In this regard, decarboxylation methodologies have been extensively advanced (26, 27). Gooβen is the leader of this progress who worked on the Cu-catalyzed decarboxylative coupling reactions to form biaryls (28-31). So far, various catalysts have been used for the decarboxylation process, including Cu(II) (29), Pd(II) (32), Ag(I) (33), and Ni(II) (34). Metal catalysis, long reaction time, difficulties in separation of catalyst, and purification of products are some drawbacks of these methods. In some cases, decarboxylation is promoted by some functional groups in the same structure (35).

**Scheme 1:**
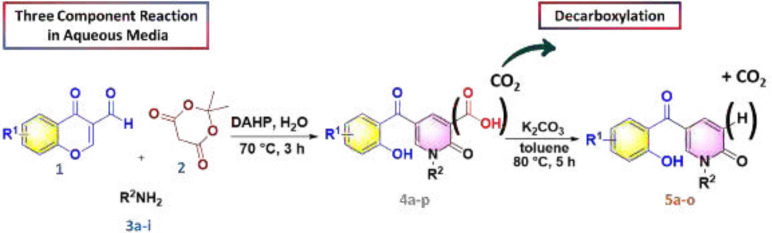
Synthesis of 2-pyridone compounds

We wish to report, herein, the preparation, evaluation of its antimicrobial properties, and molecular docking of a library of 2-pyridone-3-carboxylic acids and their decarboxylated derivatives using potassium carbonate in toluene (Scheme 1). Furthermore, *in silico* induced fit docking and molecular dynamic calculations were completed to study the spatial conformation, orientation, and interactions of these structures over *S.aureus *DNA gyrase.

## Experimental

*General*

All organic reactions were done under the air atmosphere. Commercially available substances were procured in reagent grades with high purity. Thin-layer chromatography (TLC) was aluminum plates coated with silica gel 60-F254, and the reaction progress was monitored by it under UV light of 254 nm for detection. Flash column chromatography was filled with silica gel 63-200 mesh and employed to purify products **5a-o**. The melting point was presented by the Electrothermal 9100 apparatus. ^1^H and ^13^C NMR spectroscopy were documented on a Bruker 600, 400, 300 MHz and 150, 100, 75 MHz, respectively. Chemical shifts (δ) of ^1^H and ^13^C NMR spectra were prepared as ppm, and coupling constants (*J*) have been reported in Hertz (Hz). High-resolution mass (ESI-HRMS) was obtained by Agilent Q-TOF LC-MS spectrometer.

*General process of the* ***4a-p*** *synthesis*

Products **4a-p **were prepared by the reported procedure (25).

*1-benzyl-5-(2-hydroxybenzoyl)-2-oxo-1,2-dihydropyridine-3-carboxylic acid* ***(4a)***

Yellow solid, mp 199-200 ℃, yield 86%; ^1^H NMR (400 MHz, DMSO*-d*_6_): δ_H_ = 13.51 (s, 1H, COOH), 10.43 (s, 1H, OH), 8.93 (d, *J* = 2.6 Hz, 1H, H-6-py), 8.51 (d, *J* = 2.5 Hz, 1H, H-4-py), 7.46 (t, *J* = 7.7 Hz, 1H, H-Ar), 7.41 – 7.31 (m, 6H, H-Ar), 7.02 (d, *J* = 8.2 Hz, 1H, H-Ar), 6.97 (t, *J* = 7.7 Hz, 1H, H-Ar), 5.40 (s, 2H, CH_2_N); ^13^C NMR (100 MHz, DMSO*-d*_6_): δ_C_ = 191.4, 164.9, 163.0, 156.3, 149.1, 144.8, 135.9, 133.9, 130.7, 129.2, 128.5, 128.4, 124.8, 120.0, 118.8, 117.2, 117.1, 53.6; LCMS-ESI (m/z) Calcd. for C_20_H_16_NO_5_ [M+H]^+^: 350.3500; found, 350.3.

*5-(2-hydroxybenzoyl)-2-oxo-1-(prop-2-yn-1-yl)-1,2-dihydropyridine-3-carboxylic acid* ***(4e)***

Yellow solid, mp 135-136 ℃, yield 78%; ^1^H NMR (600 MHz, CDCl_3_): δ_H_ = 13.34 (s, 1H, COOH), 11.33 (s, 1H, OH), 8.88 (d, *J* = 2.0 Hz, 1H, H-6-py), 8.65 (d, *J* = 2.0 Hz, 1H, H-4-py), 7.56 (t, *J* = 7.8 Hz, 1H, H-Ar), 7.49 (d, *J* = 7.8 Hz, 1H, H-Ar), 7.10 (d, *J* = 8.4 Hz, 1H, H-Ar), 6.95 (t, *J* = 7.8 Hz, 1H, H-Ar), 4.95 (d, 2H, *J* = 2.6 Hz, CH_2_N), 2.73 (t, *J* = 2.7 Hz, 1H, HC≡C); ^13^C NMR (151 MHz, CDCl_3_): δ_C_ = 193.8, 163.6, 163.2, 162.9, 145.6, 144.3, 137.3, 131.5, 119.5, 119.4, 119.1, 118.1, 117.0, 74.1, 79.1, 39.8; HRMS-ESI (m/z) Calcd. for C_16_H_12_NO_5_ [M+H]^+^: 298.0610; found, 298.0630.

*5-(2-hydroxybenzoyl)-2-oxo-1-((1-phenyl-1H-1,2,3-triazol-5-yl)methyl)-1,2dihydro­pyridine-carboxylic acid* ***(4g)***

White solid, mp 186-187 ℃, yield 69%; ^1^H NMR (300 MHz, CDCl_3_): δ_H_ = 13.53 (s, 1H, COOH), 11.31 (s, 1H, OH), 8.85 (d, *J* = 2.2 Hz, 1H, H-6-py), 8.68 (d, *J* = 2.1 Hz, 1H, H-4-py), 8.28 (s, 1H, CH-triazol), 7.73 (d, *J* = 7.8 Hz, 2H, H-Ar), 7.45 – 7.59 (m, 5H, H-Ar), 7.08 (d, *J* = 8.2 Hz, 1H, H-Ar), 6.96 (t, *J* = 7.6 Hz, 1H, H-Ar), 5.52 (s, 2H, CH_2_N); ^13^C NMR (75 MHz, CDCl_3_): δ_C_ = 193.8, 163.9, 163.7, 162.8, 146.4, 145.7, 140.9, 137.2, 136.6, 131.7, 129.9, 129.4, 122.6, 120.7, 119.6, 119.5, 118.9, 119.0, 117.4, 45.9; HRMS-ESI (m/z) Calcd. for C_22_H_16_N_4_NaO_5_ [M+Na]^+^: 439.1018 ; found, 439.1013.

*1-(3,4-dimethoxyphenethyl)-5-(2-hydroxybenzoyl)-2-oxo-1,2-dihydropyridine-3-carboxylic acid* ***(4h)***

 Yellow solid, mp 109-111 ℃, yield 74%; ^1^H NMR (300 MHz, CDCl_3_): 13.72 (s, 1H, COOH), 11.23 (s, 1H, OH), 8.83 (d, *J* = 2.5 Hz, 1H, H-6-py), 7.68 (d, *J* = 2.5 Hz, 1H, H-4-py), 7.46-7.53 (m, 1H, H-Ar), 7.02 (d, *J* = 8.3 Hz, 1H, H-Ar), 6.87 (dd, *J* = 8.1, 1.8 Hz, 1H, H-Ar), 6.83 (d, *J* = 7.7 Hz, 1H, H-Ar), 6.77 – 6.80 (m, 1H, H-Ar), 6.70 (d, *J* = 1.8 Hz, 1H, H-Ar), 6.58 (dd, *J* = 8.1, 1.8 Hz, 1H, H-Ar), 4.36 (t, *J* = 6.5 Hz, 2H, CH_2_N), 3.84 (s, 3H, OMe), 3.82 (s, 3H, OMe), 3.13 (t, *J* = 6.5 Hz, 2H, CH_2_Ph); ^13^C NMR (75 MHz, CDCl_3_): δ_C_ = 193.6, 164.1, 163.8, 162.7, 149.7, 148.5, 146.3, 145.4, 137.0, 131.2, 128.6, 121.3, 119.3, 118.9, 118.3, 118.0, 117.4, 111.8, 111.7, 56.0, 55.9, 54.1, 33.9; ; HRMS-ESI (m/z) Calcd. for C_23_H_22_NO_7_ [M+H]^+^ :424.1397; found, 424.1391.

*5-(2-hydroxybenzoyl)-2-oxo-1-phenyl-1,2-dihydropyridine-3-carboxylic acid* ***(4i)***

Yellow solid, mp 207-209 ℃, yield 65%; ^1^H NMR (600 MHz, CDCl_3_): δ_H_ = 13.44 (s, 1H, COOH), 11.31 (s, 1H, OH), 8.95 (d, *J* = 2.2 Hz, 1H, H-6-py), 8.28 (d, *J* = 2.1 Hz, 1H, H-4-py), 7.52 – 7.63 (m, 5H, H-Ar), 7.44 (d, *J* = 7.4 Hz, 2H, H-Ar), 7.09 (d, *J* = 8.7 Hz, 1H, H-Ar), 6.96 (t, *J* = 7.6 Hz, 1H, H-Ar); ^13^C NMR (151 MHz, CDCl_3_): δ_C_ = 193.9, 163.9, 163.8, 162.9, 146.3, 145.9, 138.4, 137.3, 131.5, 130.5, 130.0, 126.1, 119.6, 119.4, 119.1, 118.1, 117.8; HRMS-ESI (m/z) Calcd. for C_19_H_14_NO_5_ [M+H]^+^: 336.0860; found, 336.0868.

*1-benzyl-5-(5-fluoro-2-hydroxybenzoyl)-2-oxo-1,2-dihydropyridine-3-carboxylic acid* ***(4j)***

 Yellow solid, mp 186-187 ℃, yield 67%; ^1^H NMR (600 MHz, CDCl_3_): δ_H_ = 13.57 (s, 1H, COOH), 10.96 (s, 1H, OH), 8.83 (d, *J* = 2.4 Hz, 1H, H-6-py), 8.18 (d, *J* = 2.5 Hz, 1H, H-4-py), 7.89 (dd, *J* = 7.9, 3.0 Hz, 1H, H-Ar), 7.52-7.55 (m, 1H, H-Ar), 7.39 – 7.48 (m, 3H, H-Ar), 7.37 (d, *J* = 7.1 Hz, 1H, H-Ar), 7.29 – 7.24 (m, 1H, H-Ar), 7.07 – 7.00 (m, 1H, H-Ar), 5.32 (s, 2H, CH_2_N); ^13^C NMR (151 MHz, CDCl_3_): δ_C_ = 193.0, 163.9, 162.2, 161.1, 158.9, 154.7 (^1^*J*_CF_ = 241.0 Hz), 147.0, 144.8, 133.3, 129.6, 128.8, 122.9 (^2^*J*_CF_ = 25.4 Hz), 120.5 (^3^*J*_CF_ = 7.5 Hz), 118.7, 117.7, 117.0, 116.2 (^2^*J*_CF_ = 24.0 Hz), 54.0; HRMS-ESI (m/z) Calcd. for C_20_H_15_FNO_5_ [M+H]^+^: 368.0942; found, 368.0933.

*1-allyl-5-(5-fluoro-2-hydroxybenzoyl)-2-oxo-1,2-dihydropyridine-3-carboxylic acid* ***(4k)***

 Yellow solid, mp 122-123 ℃, yield 70%; ^1^H NMR (600 MHz, CDCl_3_): δ_H_ = 13.52 (s, 1H, COOH), 10.99 (s, 1H, OH), 8.83 (d, *J* = 2.1 Hz, 1H, H-6-py), 8.22 (d, *J* = 2.0 Hz, 1H, H-4-py), 7.30 (dt, *J* = 7.1, 2.8 Hz, 1H, H-Ar), 7.14 (dd, *J* = 8.3, 2.8 Hz, 1H, H-Ar), 7.07 (dd, *J* = 9.1, 4.4 Hz, 1H, H-Ar), 5.90-6.00 (ddt, *J* = 16.6, 10.2, 6.2 Hz, 1H, =CH), 5.48 (d, *J* = 10.2 Hz, 1H, =CH *cis*), 5.40 (d, *J* = 17.1 Hz, 1H, =CH *trans*), 4.78 (d, *J* = 6.1 Hz, 2H, CH_2_N); ^13^C NMR (151 MHz, CDCl_3_): δ_C_ = 193.1, 163.8, 163.7, 159.0, 154.8 (^1^*J*_CF_ = 240 Hz), 145.5, 144.8, 129.9, 124.9 (^2^*J*_CF_ = 23.6 Hz), 122.1 , 120.6 (^3^*J*_CF_ = 6 Hz), 118.8, 117.7 (^3^*J*_CF_ = 6 Hz), 117.5, 116.3 (^2^*J*_CF_ = 24 Hz), 52.9; HRMS-ESI (m/z) Calcd. For Calcd. for C_16_H_13_FNO_5 _[M+H]^+^: 318.0547; found, 318.0505.

*1-cyclohexyl-5-(5-fluoro-2-hydroxybenz oyl)-2-oxo-1,2-dihydropyridine-3-carboxylic acid* ***(4l)***

Yellow solid, mp 135-136 ℃, yield 79%; ^1^H NMR (600 MHz, CDCl_3_): δ_H_ = 13.77 (s, 1H, COOH), 11.01 (s, 1H, OH), 8.79 (s, 1H, H-6-py), 8.28 (s, 1H, H-4-py), 7.27 – 7.32 (dt, *J* = 7.1, 2.9 Hz, 1H, H-Ar), 7.14 (dd, *J* = 8.4, 2.9 Hz, 1H, H-Ar), 7.07 (dd, *J* = 9.1, 4.4 Hz, 1H, H-Ar), 4.91 – 4.98 (m, 1H, CH-cyclohexyl), 2.05 – 2.10 (m, 2H, H-cyclohexyl), 1.98 – 1.99 (m, 2H, H-cyclohexyl), 1.76– 1.82 (m, 2H, H-cyclohexyl), 1.52 – 1.58 (m, 4H, H-cyclohexyl); ^13^C NMR (151 MHz, CDCl_3_): δ_C_ = 193.5, 164.2, 163.7, 162.7, 158.9, 154.9 (^1^*J*_CF_ = 241.0 Hz), 147.0, 144.0, 124.7 (^2^*J*_CF_ = 23.5 Hz), 120.5 (^3^*J*_CF_ = 6 Hz), 118.7, 117.8 (^3^*J*_CF_ = 6 Hz), 116.3 (^2^*J*_CF_ = 23.9 Hz), 57.3, 32.6, 25.6, 25.0;; HRMS-ESI (m/z) Calcd. for C_19_H_19_FNO_5 _[M+H]^+^: 360.1242; found, 360.1251.

*5-(5-fluoro-2-hydroxybenzoyl)-2-oxo-1-(1-phenylethyl)-1,2-dihydropyridine-3-carboxylic acid* ***(4m)***

Yellow solid, mp 151-154 ℃, yield 63%; ^1^H NMR (300 MHz, CDCl_3_): δ_H_ = 13.64 (s, 1H, COOH), 10.99 (s, 1H, OH), 8.82 (d, *J* = 2.5 Hz, 1H, H-6-py), 8.03 (d, *J* = 2.5 Hz, 1H, H-4-py), 7.34 – 7.51 (m, 5H, H-Ar), 7.19 – 7.30 (m, 1H, H-Ar), 7.02 (dd, *J* = 9.1, 4.5 Hz, 1H, H-Ar), 6.83 (dd, *J* = 8.4, 2.9 Hz, 1H, H-Ar), 6.50 (q, *J* = 6.9 Hz, 1H, CHN), 1.87 (d, *J* = 7.0 Hz, 3H, CH_3_CH); ^13^C NMR (75 MHz, CDCl_3_): δ_C_ = 193.0, 163.9, 163.7, 158.9, 154.7(^1^*J*_CF_ = 241 Hz), 144.4, 143.4, 137.4, 129.7, 129.6, 127.5, 124.6 (^2^*J*_CF_ = 23.2 Hz), 120.4 (^3^*J*_CF_ = 7.5 Hz), 118.6, 117.7, 117.6, 116.1(^2^*J*_CF_ = 23.2 Hz), 56.1, 19.0; LCMS-ESI (m/z) Calcd. for C_21_H_17_FNO_5_ [M+H]^+^: 382.360 ; found, 382.2.

*1-benzyl-5-(2-hydroxy-4-methoxybenzoyl)-2-oxo-1,2-dihydropyridine-3-carboxylic acid* ***(4n)***

 Brown solid, mp 106-107 ℃, yield 57%; ^1^H NMR (300 MHz, CDCl_3_): δ_H_ = 13.42 (s, 1H, COOH), 12.04 (s, 1H, OH), 8.80 (d, *J* = 2.5 Hz, 1H, H-6-py), 8.19 (d, *J* = 2.5 Hz, 1H, H-4-py), 7.24 – 7.48 (m, 6H, H-Ar), 6.50 (d, *J* = 2.5 Hz, 1H, H-Ar), 6.42 (dd, *J* = 9.0, 2.5 Hz, 1H, H-Ar), 5.33 (s, 2H, CH_2_N), 3.86 (s, 3H, OMe); ^13^C NMR (75 MHz, CDCl_3_): δ_C_ = 192.1, 166.9, 166.2, 164.2, 163.9, 145.1, 145.0 , 133.7, 133.3, 129.5, 129.4, 128.7, 119.7, 117.2, 112.0, 108.3, 101.6, 55.8, 53.9; HRMS-ESI (m/z) Calcd. for C_21_H_18_NO_6_ [M+H]^+^: 380.1127; found, 380.1130.

*5-(2-hydroxy-4-methoxybenzoyl)-2-oxo-1-vinyl-1,2-dihydropyridine-3-carboxylic acid* ***(4o)***

Brown solid, mp 103-106 ℃, yield 52%; ^1^H NMR (300 MHz, DMSO*-d*_6_): δ_H_ = 13.77 (s, 1H, COOH), 10.98 (s, 1H, OH), 8.65 (d, *J* = 2.6 Hz, 1H, H-6-py), 8.48 (d, *J* = 2.5 Hz, 1H, H-4-py), 7.46 (d, *J* = 8.6 Hz, 1H, H-Ar), 6.50 – 6.59 (m, 2H, H-Ar), 5.96 – 6.07 (m, 1H, CHCH_2_), 5.27 (d, *J* = 9.4 Hz, 1H, =CH *cis*), 5.23 (d, *J* = 16.4 Hz, 1H, =CH *trans*), 4.79 (d, *J* = 4.8 Hz, 2H, CH_2_N), 3.80 (s, 3H, -OMe); ^13^C NMR (75 MHz, DMSO*-d*_6_): δ_C_ = 190.5, 164.5, 164.4, 162.7, 160.3, 147.8, 144.4, 133.1, 131.9, 119.0, 118.9, 116.2, 115.8, 106.8, 101.3, 55.5, 52.0; HRMS-ESI (m/z) Calcd. for C_17_H_16_NO_6_ [M+H]^+^: 330.0670; found, 330.0664.

*1-cyclohexyl-5-(2-hydroxy-4-methoxybenzoyl)-2-oxo-1,2-dihydropyridine-3-carboxylic acid* ***(4p)***

 Brown solid, mp 158-161 ℃, yield 58%; ^1^H NMR (300 MHz, CDCl_3_): δ_H_ = 13.96 (s, 1H, COOH), 12.10 (s, 1H, OH), 8.78 (d, *J* = 2.2 Hz, 1H, H-6-py), 8.26 (d, *J* = 2.2 Hz, 1H, H-4-py), 7.42 (d, *J* = 8.8 Hz, 1H, H-Ar), 6.45 – 6.56 (m, 2H, H-Ar ), 4.84 – 5.14 (m, 1H, CH- cyclohexyl), 3.89 (s, 3H, OMe), 1.89 – 2.14 (m, 4H, H-cyclohexyl), 1.83 (m, 1H, H-cyclohexyl), 1.58 (m, 4H, H-cyclohexyl), 1.18 – 1.35 (m, 1H, H-cyclohexyl); ^13^C NMR (75 MHz, CDCl_3_): δ_C_ = 192.6, 166.9, 166.2, 164.5, 163.7, 144.2, 142.2, 142.1, 133.4, 119.7, 116.5, 112.1, 108.3, 101.7, 57.1, 55.8, 32.6, 25.6, 25.0; HRMS-ESI (m/z) Calcd. for C_20_H_22_NO_6_ [M+H]^+^: 372.1292; found, 372.1295.

*General procedure for the synthesis of* ***5a-o***

5-(2-hydroxybenzoyl)-1-alkyl-2-oxo-1,2-dihydropyridine-3-carboxylic acid (0.2 mmol) and K_2_CO_3 _(0.4 mmol,55 mg) were added to an oven-dried flask containing toluene (0.5 mL) and the mixture was refluxed for 8 h. After completing the reaction based on TLC, the mixture was cooled to room temperature. The solvent was evaporated in vacuo, then DCM (15 mL) was added to the crude product and extracted from water (3 × 15 mL). The organic layer was dried over Na_2_SO_4 _and evaporated under reduced pressure. The crude product was purified by column chromatography using hexane/EtOAc 3:1 as eluent. 

*1-* *benzyl-5-(2-hydroxybenzoyl)pyridin-2(1H)-one* ***(5*** ***a*** ***)***

 White crystal, mp 120-121 ℃, yield 86%; ^1^H NMR (400 MHz, CDCl_3_): δ_H_ = 11.43 (s, 1H,OH), 7.92 (d, 1H, *J* = 2.2 Hz, H-6-py), 7.76 (dd, 1H, *J* = 9.4, 2.2 Hz, H-4-py), 7.48 (t, 1H, *J* = 7.7 Hz, H-Ar), 7.32 – 7.42 (m, 6H, H-Ar), 7.04 (d, 1H* ,J* = 8.3 Hz, H-Ar), 6.83 (t, 1H, *J* = 7.5 Hz, H-Ar), 6.66 (d, 1H, *J* = 9.5 Hz, H-3-py), 5.19 (s, 2H, –CH_2_N); ^13^C NMR (100 MHz, CDCl_3_): δ_C_ = 195.1, 162.4, 162.1, 143.2, 139.0, 136.2, 135.3, 131.5, 129.2, 128.6, 128.5, 120.2, 118.8, 118.9, 118.7, 117.3, 52.6; HRMS-ESI (m/z) Calcd. for C_19_H_16_NO_3 _[M+H]^+^: 306.1135; found, 306.1138.

*1-allyl-5-(2-hydroxybenzoyl)pyridin-2(1H)-one* ***(5b)***

 White crystal, mp 128-129 ℃, yield 88%; ^1^H NMR (300 MHz, CDCl_3_): δ_H_ = 11.43 (s, 1H, OH), 7.93 (d, 1H, *J* = 2.3 Hz, H-6-py), 7.75 (dd, 1H, *J* = 9.5, 2.6 Hz, H-4-py), 7.44 – 7.59 (m, 2H, , H-Ar), 7.08 (d, 1H, *J* = 8.3 Hz, H-Ar), 6.93 (t, 1H, *J* = 7.6 Hz, H-Ar), 6.64 (d, 1H, *J* = 9.5 Hz, H-3-py), 5.91 – 6.06 (m, 1H, CHCH_2_), 5.36 (d, 1H, *J* = 10.1 Hz, CH_2_=CH *cis*), 5.29 (d, 1H, *J* = 17.1 Hz, CH_2_=CH *trans*), 4.64 (d, 2H, *J* = 5.8 Hz, CH_2_N); ^13^C NMR (75 MHz, CDCl_3_): δ_C_ = 195.1, 162.5, 161.7, 142.9, 139.0, 136.5, 131.5, 120.1, 120.0, 119.8, 118.9, 118.8, 118.7, 117.3, 51.7; HRMS-ESI (m/z) Calcd. for C_15_H_14_NO_3 _[M+H]^+^: 256.0960; found, 256.0964.

*1-cyclohexyl-5-(2-hydroxybenzoyl)pyridin-2(1H)-one* ***(5c)***

 Yellow crystal, mp 115-116 ℃, yield 83%; ^1^H NMR (300 MHz, CDCl_3_): δ_H_ = 11.49 (s, 1H, OH), 8.00 (d, 1H*, J* = 2.5 Hz, H-6-py), 7.73 (dd, 1H, *J* = 9.5, 2.6 Hz, H-4-py), 7.50 – 7.60 (m, 2H, H-Ar), 7.08 (d, 1H, *J* = 7.8 Hz, H-Ar), 6.95 (dt, 1H, *J* = 8.4, 1.2 Hz, H-Ar), 6.61 (d, 1H* , J* = 9.5 Hz, H-3-py), 4.91 (m, 1H, CH, CH- cyclohexyl), 2.04 – 1.36 (m, 10H, H-cyclohexyl);^13^C NMR (75 MHz, CDCl_3_): δ_C_ = 195.4, 162.5, 161.7, 139.9, 138.1, 136.0, 131.5, 119.6, 118.9, 118.8, 118.7, 117.1, 54.8, 32.6, 25.7, 25.2; HRMS-ESI (m/z) Calcd. for C_18_H_20_NO_3 _[M+H]^+^: 298.1540; found, 298.1533.

*1-(furan-2-ylmethyl)-5-(2-hydroxybenzoyl)pyridin-2(1H)-one* ***(5d)***

 Yellow solid, mp 127-128 ℃, yield 68%; ^1^H NMR (400 MHz, CDCl_3_): δ_H_ = 11.46 (s, 1H, OH), 7.99 (d, 1H* ,J* = 2.2 Hz, H-6-py), 7.77 (dd, 1H, *J* = 9.5, 2.4 Hz, H-4-py), 7.50 (t, 2H, *J* = 8.0 Hz, H-Ar), 7.43 (d, 1H, *J* = 1.8 Hz, H-furyl ), 7.07 (d, 1H, *J* = 8.2 Hz, H-Ar), 6.90 (t, 1H, *J* = 7.6 Hz, H-Ar), 6.64 (d, 1H, *J* = 9.5 Hz, H-3-py), 6.51 (d, 1H, *J* = 3.0 Hz, H-furyl), 6.36 – 6.42 (m, 1H, H-furyl), 5.18 (s, 2H, CH_2_N);^13^C NMR (100 MHz, CDCl_3_): δ_C_ =195.1, 162.5, 161.7, 147.8, 143.6, 142.9, 139.1, 136.2, 131.7, 120.2, 118.9, 118.8, 118.7, 117.3, 111.1, 111.0, 45.1; HRMS-ESI (m/z) Calcd. for C_17_H_14_NO_4_ [M+H]^+^: 296.0931; found, 296.0921.

*5-* *(2-hydroxybenzoyl)-1-(prop-2-yn-1-yl)pyridin-2(1H)-one* ***(5e)***

White solid, mp 117-116 ℃, yield 68%; ^1^H NMR (400 MHz, CDCl_3_): δ_H_ = 11.46 (s, 1H, OH), 8.35 (d, 1H, *J* = 2.4 Hz , H-6-py), 7.82 (dd, 1H* , J* = 9.5, 2.5 Hz, H-4-py), 7.56 – 7.62 (m, 1H, H-Ar), 7.56 – 7.49 (m, 1H, H-Ar), 7.09 (d, 1H*, J* = 8.3 Hz,H-Ar), 6.94 (t, 1H, *J* = 7.5 Hz, H-Ar), 6.65 (d, 1H* , J* = 9.5 Hz, H-3-py), 4.83 (d, 2H, *J* = 2.5 Hz, CH_2_N), 2.61 (t, 1H, *J* = 2.5 Hz, CH≡C); ^13^C NMR (100 MHz, CDCl_3_): δ_C_ = 195.1, 162.4, 162.1, 141.7, 139.4, 136.7, 136.3, 131.6, 129.0, 119.7, 118.9, 118.8, 117.5, 75.8, 38.5; HRMS-ESI (m/z) Calcd. for C_15_H_12_NO_3_ [M+H]^+^: 254.0610; found, 254.0601.

*5-(2-hydroxybenzoyl)-1-(1-phenylethyl)pyridin-2(1H)-one* ***(5f)***

 Yellow solid, mp 95-97 ℃, yield 87%; ^1^H NMR (400 MHz, CDCl_3_): δ_H_ = 11.42 (s, 1H, OH), 7.77 (d, 1H, *J* = 2.5 Hz, , H-6-py), 7.70 – 7.73 (m, 1H , H-4-py ), 7.33 – 7.48 (m, 6H, H-Ar), 7.11 (dd, 1H* , J* = 8.0, 1.4 Hz, H-Ar), 7.01 (d, 1H* , J* = 8.0 Hz, H-Ar), 6.68 (t, 2H, *J* = 8.0 Hz, H-Ar, H-3-py )**,** 6.46 (q, 1H, *J* = 7.0 Hz, CHN), 1.74 (d, 3H, *J* = 7.0 Hz, CH_3_); ^13^C NMR (100 MHz, CDCl_3_): δ_C_ = 194.9, 162.4, 161.8, 141.1, 139.2, 138.2, 135.9, 131.3, 129.2, 128.5, 127.5, 120.0, 118.7, 118.6, 118.5, 117.1, 53.7, 18.9; HRMS-ESI (m/z) Calcd. for C_20_H_18_NO_3_ [M+H]^+^: 320.1393; found, 320.1397.

*1-* *benzyl-5-(5-fluoro-2-hydroxybenzoyl)pyridin-2(1H)-one* ***(5*** ***j*** ***)***

 Yellow solid, mp 105-106 ℃, yield 84%; ^1^H NMR (300 MHz, CDCl_3_): δ_H_ = 11.08 (s, 1H, OH), 7.93 (d, 1H, *J* = 2.4 Hz, H-6-py), 7.76 (dd, 1H, *J* = 9.5, 2.6 Hz, , H-4-py), 7.32 – 7.45 (m, 5H, H-Ar), 7.19 – 7.27 (m, 1H, H-Ar), 7.08 (dd, 1H, *J* = 8.7, 3.0 Hz, H-Ar), 7.02 (dd, 1H, *J* = 9.1, 4.5 Hz, H-Ar), 6.68 (d, 1H, *J* = 9.5 Hz, , H-3-py), 5.20 (s, 2H, CH_2_N); ^13^C NMR (75 MHz, CDCl_3_): δ_C_ = 194.0, 161.9, 158.5, 154.6 (^1^*J*_CF_ = 240.0 Hz), 143.2, 138.5, 135.1, 129.3, 128.7, 128.5, 123.5 (^2^*J*_CF_ = 23.6 Hz), 120.4, 120.0 (^3^*J*_CF_ = 6.0 Hz), 118.3 (^3^*J*_CF_ = 6.1 Hz), 116.8, 116.4 (^2^*J*_CF_ = 24.0 Hz), 52.6; HRMS-ESI (m/z) Calcd. for C_19_H_15_FNO_3_ [M+H]^+^: 324.1014; found, 324.1012.

*1-allyl-5-(5-fluoro-2-hydroxybenzoyl)pyridin-2(1H)-one* ***(5k)***

 Yellow solid, mp 100-101 ℃, yield 78%; ^1^H NMR (300 MHz, CDCl_3_): δ_H_ = 11.12 (s, 1H, OH), 7.96 (d, 1H* , J* = 2.5 Hz, H-6-py), 7.77 (dd, 1H, *J* = 9.5, 2.5 Hz, H-4-py), 7.16 – 7.33 (m, 2H, H-Ar), 7.06 (dd, 1H, *J* = 8.9, 4.6 Hz, H-Ar), 6.66 (d, 1H, *J* = 9.5 Hz, H-3-py), 5.89 – 6.09 (m, 1H, CHCH_2_), 5.35 – 5.41 (m, 1H, =CH *cis*), 5.30 (dd, 1H, *J* = 16.0, 0.9 Hz, =CH *trans*), 4.65 (d, 2H, *J* = 6.0 Hz, CH_2_N); ^13^C NMR (75 MHz, CDCl_3_): δ_C_ = 194.1, 161.6, 158.5, 154.7 (^1^*J*_CF_ = 239.5 Hz), 143.1, 138.6, 131.5, 131.4, 123.6 (^2^*J*_CF_ = 23.7 Hz), 120.2, 120.1 (^3^*J*_CF_ = 6.5 Hz), 118.4 (^3^*J*_CF_ = 6.4 Hz), 116.8, 116.5 (^2^*J*_CF_ = 24.3 Hz), 51.7; HRMS-ESI (m/z) Calcd. for C_15_H_13_FNO_3_ [M+H]^+^: 274.0825; found, 274.0820.

*1-* *cyclohexyl-5-(5-fluoro-2-hydroxybenz-oyl)pyridin-2(1H)-one* ***(5*** ***l*** ***)***

 Yellow solid, mp 125-126 ℃, yield 78%; ^1^H NMR (300 MHz, CDCl_3_): δ_H_ = 11.17 (s, 1H, OH), 8.02 (d, 1H, *J* = 2.5 Hz, H-6-py), 7.72 (dd, 1H*, J* = 9.5, 2.6 Hz, H-4-py), 7.18 – 7.34 (m, 2H, H-Ar), 7.06 (dd, 1H, *J* = 9.0, 4.6 Hz, H-Ar), 6.62 (d, 1H, *J* = 9.5 Hz, H-3-py), 4.81 – 4.98 (m, 1H, CH- cyclohexyl), 1.11 – 2.07 (m, 10H, H-cyclohexyl); ^13^C NMR (75 MHz, CDCl_3_): δ_C_ = 194.3, 161.6, 158.6, 158.5, 154.7 (^1^*J*_CF_ = 239.6 Hz), 140.1, 137.8, 123.4 (^2^*J*_CF_ = 23.6 Hz), 120.1 (^3^*J*_CF_ = 6.0 Hz), 119.7, 118.5 (^3^*J*_CF_ = 6.3 Hz), 116.5 (^2^*J*_CF_ = 23.8 Hz), 54.9, 32.6, 25.7, 25.2; HRMS-ESI (m/z) Calcd. for C_18_H_19_FNO_3_ [M+H]^+^: 316.1212; found, 316.1207.

*1-benzyl-5-(2-hydroxy-4-methoxybenzoyl)pyridin-2(1H)-one* ***(5o)***

 Brown solid, mp 83-85 ℃, yield 60%; ^1^H NMR (400 MHz, CDCl_3_): δ_H_ = 12.20 (s, 1H, OH), 7.86 (d, 1H,* J* = 2 Hz, H-6-py), 7.71 (dd, 1H, *J* = 9.5, 2.2 Hz, H-4-py), 7.29 – 7.43 (m, 6H, H-Ar), 6.66 (d, 1H, *J* = 9.5 Hz, H-3-py), 6.49 (d, 1H, *J* = 2.1 Hz, H-Ar), 6.37 (dd, 1H* , J* = 8.9, 2.2 Hz, H-Ar), 5.20 (s, 2H, CH_2_N), 3.85 (s, 3H, OCH_3_); ^13^C NMR (100 MHz, CDCl_3_): δ_C_= 193.7, 166.2, 165.8, 162.1, 142.2, 139.0, 135.4, 133.3, 129.2, 128.6, 128.5, 120.2, 117.7, 112.5, 107.6, 101.4, 55.7, 52.5; HRMS-ESI (m/z) Calcd. for C_20_H_18_NO_4_ [M+H]^+^: 336.1015; found, 336.1010.

*General procedure for the synthesis of 1-benzyl-2-oxo-1,2-dihydropyridine-3-carbo-xylic acid* ***(6)*** (36)

Compound 2-chloro-1,2-dihydropyridine-3-carboxylic acid (1 mmol) in 70% aqueous acetic acid (10 mL) was heated under reflux for 4-6 h (see ref 5 in article). Completion of the reaction was checked by TLC. After cooling a solid product precipitated which was filtered, washed well with water, dried and purified by recrystallization from DMF. Compound 2-oxo-1,2-dihydropyridine-3-carboxylic acid (1 equiv) in DMF (4 mL) BnBr (1.3 equiv) and potassium carbonate (1.5 equiv) were added. The reaction mixture was stirred at room temperature during 20-50 h. After completion (checked by TLC) the reaction mixture was poured into ice-water (25 mL) and the solid product formed was filtered, washed well with water, dried and purified by recrystallization from EtOH.

*1-benzyl-2-oxo-1,2-dihydropyridine-3-carboxylic acid* ***(6)***

 White solid, mp 128-130 ℃, yield 67%; ^1^H NMR (300 MHz, DMSO*-d*_6_): δ_H_ = 14.46 (s, 1H, COOH), 8.42-8.36 (m, 2H, H-4,6-py), 7.40-7.27 (m, 5H, Bn), 6.76 (t, *J* = 6.8 Hz, 1H, H-5-py), 5.31 (s, 2H, -CH_2_); ^13^C NMR (75 MHz, , DMSO*-d*_6_): δ_C_ = 164.72, 163.6, 145.6, 145.1, 135.8, 128.8, 128.1, 127.9, 116.9, 108.8, 52.6.

*General procedure for the preparation of Methyl 1-benzyl-5-(2-hydroxybenzoyl)-2-oxo-1,2-dihydropyridine-3-carboxylate* ***(7)***

 Compound 4a (1mmol) in DMF (4 mL) were added methyl iodide (1 mmol) and potassium carbonate (1.5 mmol). The reaction mixture was stirred at rt for 2.5 days. Water was added and the reaction mixture was extracted with hexanes-ether (1:1) solvent system. The combined organic phases were dried over Na_2_SO_4_, filtered, and concentrated in a vacuum to yield 8a (40%) as an orange solid. 

Red solid, mp 150-152 ℃, yield 60%; ^1^HNMR (300 MHz, CDCl_3_): δ_H_ = 8.49 (d, *J* = 2.6 Hz, 1H, H-6-py), 8.16 (d, *J* = 2.6 Hz, 1H, H-4-py), 7.45 (t, *J* = 7.5 Hz, 1H, H-Ar), 7.36-7.29 (m, 6H, H-Ar), 7.01 (t, *J* = 7.5 Hz, 1H, H-Ar), 6.91 (d, *J* = 8.4 Hz, 1H, H-Ar), 5.15 (s, 2H, CH_2_N), 3.85 (s, 3H, CH_3_), 3.60 (s, 3H, CH_3_), ^13^CNMR (75 MHz, CDCl_3_): δ_C_ = 190.4, 165.0, 159.0, 156.6, 147.3, 144.2, 134.9, 132.8, 129.8, 129.0, 128.5, 126.8, 121.1, 119.3, 116.8, 111.3, 55.4, 53.3, 53.4; HRMS-ESI (m/z) C_22_H_20_NO_5 _[M+H]^+^ : 378.1341; found, 378.1349.

*Ethyl 1-benzyl-5-(2-hydroxybenzoyl)-2-oxo-1,2-dihydropyridine-3-carboxylate* ***(8)***

White solid, mp 115-117 ℃, yield 73%; ^1^HNMR (600 MHz, CDCl_3_): δ_H_ = 11.38 (s, 1H, OH), 8.53 (s, 1H, H-6-py), 8.14 (s, 1H, H-4-py), 7.53 (t, *J* = 7.6 Hz, 1H, H-Ar), 7.43 – 7.37 (m, 6H, H-Ar), 7.09 (d, *J* = 8.4 Hz, 1H, H-Ar), 6.88 (t, *J* = 7.7 Hz, 1H, H-Ar), 5.26 (s, 2H, CH_2_N), 4.42 (q, *J* = 7.2 Hz, 2H, CH_2_O), 1.41 (t, *J* = 7.2 Hz, 3H, CH_3_CH_2_); ^13^CNMR (151 MHz, CDCl_3_): δ_C_ = 194.3, 164.1, 162.6, 158.7, 146.1, 143.6, 136.5, 134.8, 131.4, 129.3, 128.9, 128.8, 120.5, 119.0, 118.9, 118.5, 115.9, 61.7, 53.3; HRMS-ESI (m/z) C_22_H_20_NO_5 _[M+H]^+^ : 378.1278; found, 378.1273.

*Antimicrobial assay*

All new structures were assessed to *in vitro* tests for their antibacterial properties. The antibacterial activities were evaluated against *E. coli* PTCC 25922,* A.* *baumannii *(clinical strain); *S. aureus *ATCC 1431; *C. albicans* ATCC 10231 and Methicillin-resistant *S. aureus* (clinical strain). 

The values of minimum inhibitory concentration (MIC) were assayed by the standard technique described by CLSI (37) meaning standard broth micro-dilution procedure.

The culture of each strain was prepared on a nutrient agar plate and remained overnight, then, it was used to make the bacterial suspensions equal to 0.5 McFarland standards in sterile normal saline. Then Serial dilutions of samples were obtained in sterile 96 wells plates containing Mueller–Hinton broth which was prepared with concentrations ranging from 0.031 to 64 mg/ml. The final concentration of bacterial cells in each well was 0.5–1 ×10^6^ cfu mL^-1^ approximately.

Minimum inhibitory concentrations (MICs) were recorded after 22 hours of incubation at 37 ºC. Each experiment was performed in triplicate. Cefixime, Ciprofloxacin and Nystatin were used as the reference antibiotics for bacteria and yeast, respectively.

*Molecular modeling procedure*

*Target enzyme and ligand preparation*

To find out the interactions mode of designed molecules over DNA gyrase, the Maestro Molecular Modeling platform (version 10.5) by Schrödinger, LLC was performed (38). The X-ray crystallographic structure of *S. aureus* DNA gyrase (in complex with moxifloxacin and DNA) was downloaded from the Protein Data Bank (PDB ID; 5cdq) (www.rcsb.org) (39).

As urease is reported to be functionally active in a monomeric state, all the docking studies were performed on a single monomer. In addition, prosthetic group and co-factors are not directly involved in urease inhibition, so they were removed before docking investigation. Water molecules and co-crystallized ligands were removed from the enzyme’s crystallographic structures. 

The 2D structures of all synthesized compounds were drawn in Marvin 15.10.12.0 program (http://www.chemaxon.com) (40) and converted into a pdb file. The Protein Preparation Wizard (41) and the LigPrep (42) module were used to prepare protein and ligand structure properly. The missing side chains of the proteins were filled using the Prime tool and missing residues were updated. 

*Induced fit docking (IFD) protocol*

IFD method implemented in Glide software (Schrödinger LLC 2018, USA) was used to investigate the possible binding mode of the most active inhibitors in the active site of DNA gyrase (38). The moxifloxacin binding site was used to generate the grid for IFD calculation. The maximum 20 poses with receptor and ligand van der Waals radii of 0.7 and 0.5, respectively considered. Residues within 5 Å of the moxifloxacin at the active site were refined followed by side-chain optimization. Structures whose Prime energy is more than 30 kcal/mol are eliminated based on extra precious Glide docking. 

*Molecular dynamic (MD) simulation*

Molecular simulations of this study were performed using the Desmond v5.3 using the Maestro interface (from Schrödinger 2018‐4 suite) (43). The appropriate pose for the MD simulation procedure of the compound was achieved by the IFD method. To build the system for MD simulation, the protein-ligand complexes were solvated with SPC explicit water molecules and placed in the center of an orthorhombic box of appropriate size in the periodic boundary condition. Sufficient counter‐ions and a 0.15 M solution of NaCl were also utilized to neutralize the system and to simulate the real cellular ionic concentrations, respectively. The MD protocol involved minimization, pre-production, and finally production MD simulation steps. In the minimization procedure, the entire system was allowed to relax for 2500 steps by the steepest descent approach. Then the temperature of the system was raised from 0 to 300 K with a small force constant on the enzyme to restrict any drastic changes. MD simulations were performed via NPT (constant number of atoms, constant pressure *i.e*. 1.01325 bar and constant temperature *i.e*. 300 K) ensemble. The Nose‐Hoover chain method was used as the default thermostat with 1.0 ps interval and Martyna‐Tobias‐Klein as the default barostat with 2.0 ps interval by applying an isotropic coupling style. Long‐range electrostatic forces were calculated based on the particle‐mesh‐based Ewald approach with the cut‐off radius for columbic forces set to 9.0 Å. Finally, the system was subjected to produce MD simulations for 20 ns for each protein-ligand complex. During the simulation was stored every 1000 ps of the actual frame. The dynamic behavior and structural changes of the systems were analyzed by the calculation of the root mean square deviation (RMSD) and RMSF. Subsequently, the energy-minimized structure calculated from the equilibrated trajectory system was evaluated for the investigation of each ligand-protein complex interaction.

*In-silico ADME properties of synthesized compounds*

QikProp module of Schrodinger was applied to calculate the important pharmacokinetic properties of the synthesized compounds like drug-likeness, metabolism and cell permeation (44).

## Results and Discussion

*Synthesis and chemistry*

**Scheme 2:**
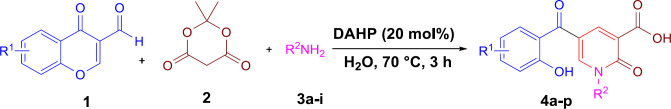
Synthesis of 2-pyridone-3-carboxylic acids

According to our previously published research (25), at first 2-pyridone-3-carboxylic acid **4a** was synthesized through a three-component reaction of 3-formylchromone **1**, Meldrum’s acid **2**, and benzylamine **3a**. To find the best reaction conditions, diammonium hydrogen phosphate (DAHP) was examined as a basic catalyst of this reaction at different temperatures in different solvents. Correspondingly, as shown in Scheme 1, DAHP 20 mol% in water at 70 °C was selected as the optimized reaction conditions (Scheme 2) to develop the diversity of the products **4a-p** by the use of various aliphatic or aromatic amines **3a-i** (Table 1). Compared to our previous publication (25), in the current study, some variations were applied including the use of different amines having propargyl, triazole and phenyl rings, and 3-formyl-chromenes having OMe and F substitution on its ring.

**Scheme 3:**
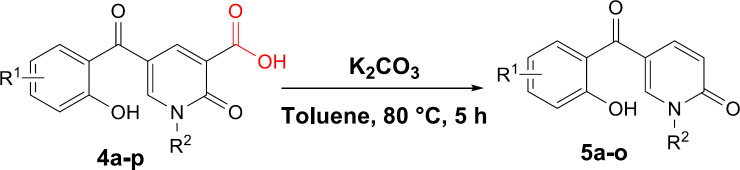
Decarboxylation reaction of 2-pyridone-3-carboxylic acids

Next, we focused on the decarboxylation of the prepared 2-pyridone-3-carboxylic acids **4a-p**. Compound **4a **was chosen for the reaction optimization. Solvent, temperature, and the base were systematically varied (Table 2). Among the tested bases (entries 1-6), K_2_CO_3_ and Ag_2_CO_3_ gave the highest yields of 46 and 56%, respectively. According to the efficient amount, cost and availability, K_2_CO_3_ was used to find the solvent and the optimized heating. Correspondingly, the best result was attained using K_2_CO_3_ as the base by increasing its amounts and running the reaction in toluene at 80 °C for 5 hours to afford the decarboxylated 2-pyridone **5a **in 86% yield (entry 10). Therefore, some 2-pyridone-3-carboxylic acids **4a-p **were treated in these conditions (Scheme 3), and their corresponding decarboxylated products were produced in moderate to high yields (Table 3). 

**Scheme 4:**
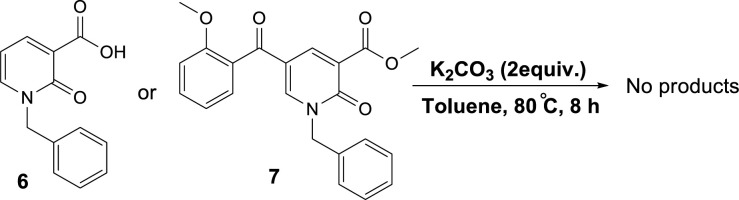
Control reaction to confirm the role of the hydroxyl group in decarboxylation

**Scheme 5:**
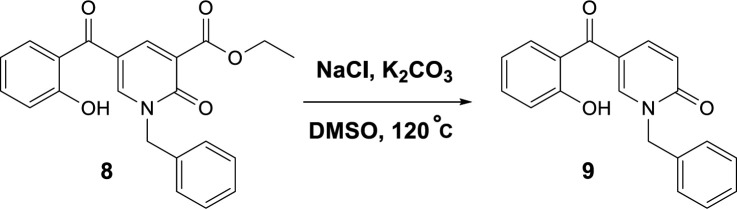
Control reaction to confirm Krapcho mechanism

To propose a reasonable reaction mechanism, we used two 2-pyridone derivatives **6** and **7** as substrates in decarboxylation reaction. The effective role of the hydroxyl group through the decarboxylation reaction was studied using the deoxy derivative of 1-benzyl-2-oxo-1,2-dihydropyridine-3-carboxylic acid **6** and OH-protected compound of methyl 1-benzyl-5-(2-methoxybenzoyl)-2-oxo-1,2-dihydropyridine-3-carboxylate **7** (Scheme 4), and no decarboxylated product was detected confirming the hydroxyl group is important for decarboxylation process. Thus, we concluded that this reaction may proceed via Krapcho decarboxylation (45). To confirm our proposed mechanism passing from Krapcho decarboxylation, ethyl 1-benzyl-5-(2-hydroxybenzoyl)-2-oxo-1,2-dihydropyridine-3-carboxylate **8** was treated under the optimized conditions (Scheme 5), and the desired product **9** was isolated in 30% yield. This demonstrates that both carboxylic acid and ester groups on 2-pyridone are decarboxylated in the presence of K_2_CO_3_ that is because of an electron-withdrawing group in the beta position (Krapcho condition).

**Scheme 6:**
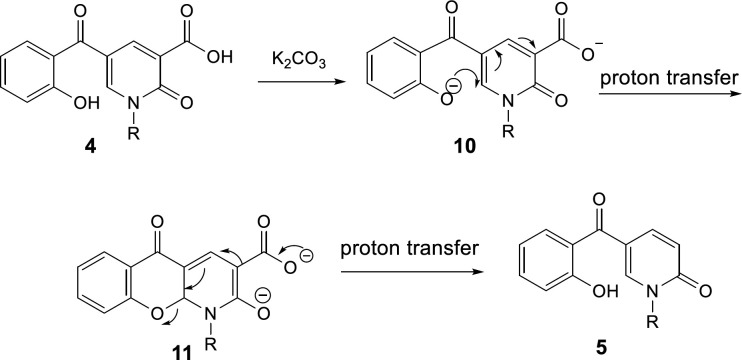
The plausible mechanism for decarboxylation of 2-pyridone-3-carboxylic acids

A plausible mechanism of the decarboxylation of 2-pyridone-3-carboxylic acids has been shown in Scheme 6. K_2_CO_3_ acts as the base to react with the protons of hydroxyl and carboxylic acid groups in compound **4**, then, oxa-Michael addition of phenoxy anion **10** gives intermediate **11**. Since toluene solvent is not dry, its moisture accelerates the proton transfer to promote this reaction. Furthermore, because just one molecule is divided into two, it increases the entropy accompanying by releasing heat, thus elimination of CO_2_ is a preferable driving force. Consequently, intermediate **11** can participate in Krapcho decarboxylation (45), passing from **12**, C-O bond is broken following protonation the decarboxylated product **5** is formed. 

*Biological activity evaluation*

*Antimicrobial assay*

In this study, the antimicrobial activity of compounds was assessed against two Gram-positive (*S. aureus *PTCC 1431 and *Methicillin-resistant S. aureus (MRSA)* (clinical strain) and two Gram-negative bacteria strains* (E. coli* PTCC 25922 and* A.* *baumannii *(clinical strain) and *C. albicans* ATCC 10231. The MIC values of the screened compounds were compared with cefixime, ciprofloxacin, and nystatin as the standard antibacterial agents and listed in Table 4. 

In the case of Gram-negative bacteria, compound **4h** showed the best antibacterial activity against *E. coli *with MIC value of 12.79 µg/mL and compounds **5f** and **5j **were showed moderate anti-*E. coli* effect with MIC value of 159.68 and 161.66 µg/mL, respectively.

Furthermore, compounds **4m**, **4n**, **4o**, **5b**, **5c** and **5d **recorded moderate inhibitory effects against *Acinetobacter*, which is one of the most important human pathogenic. 

Based on the result, it was observed that the synthesized compound has higher inhibition activity over *S. aureus. *In other words, compounds **4b**, **4j**, **4n**,** 4o**,** 4p**, **5a**, **5b** and **5c** represented significant higher antibacterial activity against *S. aureus* strain with MIC values of 4.67, 5.73, 5.92, 10.29, 2.32, 9.5, 3.2 and 2.32 µg/mL, respectively. 

As can be seen, none of the compounds showed comparable or more activity determined by *C. albicans* against nystatin, and only compounds **5c** and **5l **with MIC values of 279.32 and 315.34 µg/ml, respectively, revealed better results than the other compounds. The introduction of the 4-methoxy substituent at the R^2^ position of the template structure with carboxylic acid led to a significant increase in the antibacterial activity of this compound against *S. aureus* like compounds **4n**, **4o**, and **4p**.

*Reliability of the docking protocol*

**Figure 2:**
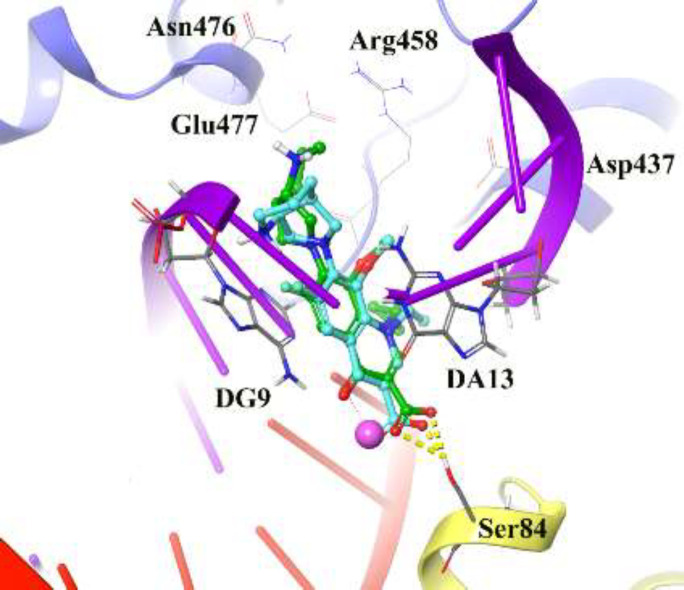
3D representation of moxifloxacin over DNA gyrase active site. Co-crystallized moxifloxacin and the corresponding re-docked form represented in green and cyan color, respectively. GyrA and GyrB subunit of DNA gyrase are in yellow and blue, respectively

The reliability of the applied docking protocol was assessed by re-docking of moxifloxacin into the active site of DNA gyrase. The RMSD of the docked binding mode compared with the crystallographic binding mode. The reliability of the docking procedure was considered successful if the RMSD is below 2.0 Å. Figure 2 shows the superposed structures between the docked and the crystallographic moxifloxacin over DNA gyrase, which has an acceptable value within the cutoff limit (2.23 Å). Then, this protocol was similarly applied to all synthesized compounds (**4a**-**4p** and **5a**-**5o**).

Figure 2 depicts the docked moxifloxacin intercalates into DNA at the nicks introduced by the topoisomerases and that the pyrrolo[3,4-*b*]pyridine moiety at C-7 of ring system interacts with the GyrB subunit. The distal nitrogen atom of the pyrrolo[3,4-*b*]pyridine moiety at the C-7 ring system lies close to Glu477 in *S. aureus *GyrB, whereas the 3-carboxyl end of the moxifloxacin interacted to Ser84 located at the GyrA subunit, through a magnesium-water bridge hydrogen bond with 1.4 Å distance that stabilizes the drug-enzyme DNA complex (46). In summary, Moxifloxacin bounds in the DNA at the cleavage sites making specific interactions with the protein and inhibiting DNA relegation (47).

*Investigating molecular docking interaction of the synthesized compounds*

The performed docking procedure was then applied to evaluate the interactions between newly synthesized compounds; **4a**-**4p** and **5a**-**5o **over the DNA gyrase active site. The top IFD scoring pose of all compounds was analyzed inside the binding site of DNA gyrase. 

**Figure 3:**
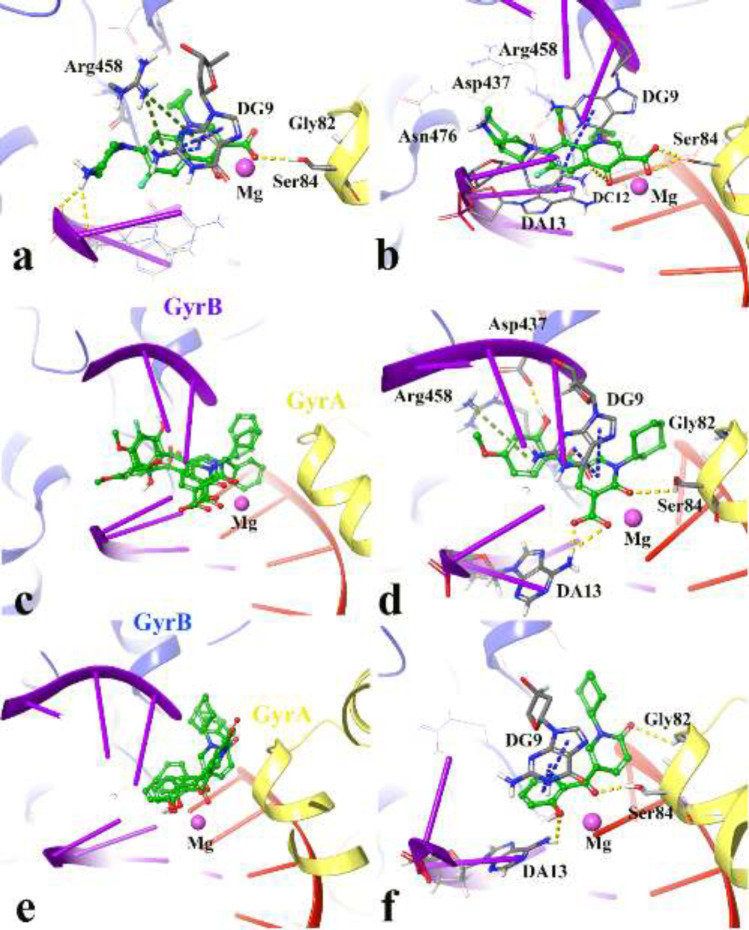
3D representation of the modeled cleaved complex consisting of the best IFD pose interaction of compounds over DNA gyrase. The studied compounds include; ciprofloxacin (a), moxifloxacin (b) the superposed compounds **4j**, **4n**, **4p** (c), compound **4p** (d), the superposed compounds **5a**, **5b**, **5c** (e), and compound **5c **(f). GyrA and GyrB subunit of DNA gyrase are in yellow and blue, respectively

Figures 3a and 3b show the quinolone ring moiety of ciprofloxacin and moxifloxacin intercalated between the DNA base pairs through π-π hydrophobic interaction with DG9 and DA13. In addition, the 4-oxo-3-carboxylic acid groups of the quinolone nucleus tightly coordinated along with the Mg ion and further stabilized by H-bond interaction with Ser84 (with 1.6 Å and 1.2 Å, respectively) located at the α-helix IV of GyrA while the tail part at the C-7 of the quinolone ring system of both compounds (7-piperazine and 7-pyrrolo[3,4-*b*]pyridin group) faced to the GyrB subunit.

The docked conformation of compounds **4j**, **4n**, **4p** revealed that they all have the same orientation in which they intercalated into the DNA and pointed toward the Mg ion through 2-oxo and 3-carboxylic groups. Furthermore, the N-alkyl moiety faced the GyrA α-helix (yellow helix), while the 3,4-disubstituted phenol ring closed to the GyrB subunit (blue helix) (Figure 3c). 

Figure 3d represents the cross-link orientation of compound **4p** over the cleaved complex of DNA gyrase, in which the 2-hydroxy-4-methoxybenzoyl interacted with Arg458 and Asp437 through π-cation and H-bond, respectively, while the bulky N-cyclohexane and the 4-carbonyl groups occupied in the proximity of Gly81 and formed H-bond interaction with Ser84, (1.6 Å), respectively. Comparing the orientation of compound **4p** with the well-known inhibitors (Figures 3a and 3b), revealed that the 2-hydroxy-4-methoxybenzoyl has the same location and orientation as the 7-piperazine and 7-pyrrolo[3,4-*b*]pyridin groups of ciprofloxacin and moxifloxacin, respectively. In addition, the electron-donating property of the methoxy substitution at the benzoyl ring increases the tendency of the compound **4p** to make π-cation interaction with Arg458 in which stabilized the cleaved complex. This may propose the reason why compounds **4n**, **4o**, and **4p** with 4-methoxy group represent higher *S. aureus* inhibition activity.

Same as the compounds in the 4^th^ series, compounds **5a**, **5b**, and **5c **overlay in an identical location and orientation and stabilized the cleaved complex through cross-linking the GyrA and GyrB subunits by intercalating the DNA fragment (Figure 3e). Although the 3-carboxyl group was omitted, the hydroxyl and carbonyl groups of 2-hydroxybenzoyl moiety oriented toward the Mg ion and further stabilized to the GyrA through H-bond interaction with Ser84. As a result of Figure 3f observation, the 2-pyridone ring closed to the GyrA α-helix (yellow helix) and formed H-bond with Gly82 (2.4 Å), while the related N-alkyl group twisted back and faced toward the GyrB subunit (blue helix) surrounded by Ser438 and Gly436. In summary, the docking results revealed the mentioned active compounds successfully intercalated the DNA gate region of the cleaved complex through cross-linking the GyrA and GyrB subunits of DNA gyrase like as ciprofloxacin and moxifloxacin.

*Molecular dynamic investigation*

To understand the criteria for rational designing of gyrase inhibitors, it is necessary to uncover the structural perturbations incurred by the most potent compounds (**4p**, **5c**) over DNA gyrase and the effect of these compounds on the DNA gate environment in comparison to ciprofloxacin as DNA gyrase standard inhibitor.

**Figure 4:**
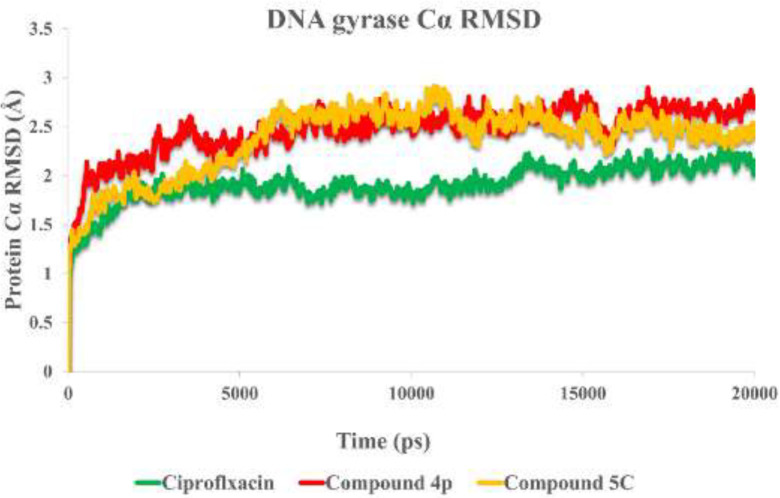
RMSD of the DNA gyrase Cα in complexed with ciprofloxacin (in green) compounds **4p** (in red) and compound **5c** (in orange) over 20 ns MD simulation time

Root mean square deviation (RMSD) of the protein’s Cα from its initial to final conformation applied over 20 ns MD simulation to study the stability of the protein-ligand complex. The RMSD values of gyrase-ligand complexes indicate that the employed simulation time has been enough to obtain an equilibrium structure (Figure 4). Thus, the structure at the MD equilibrium state was used to investigate the structural specificity of the ligand-protein complexes. The RMSD simulation showed that gyrase makes a complex with ciprofloxacin maintained overall stability after 5 ns of MD simulation time with higher ﬂuctuation stabilizing at an average of 2 Å (Figure 4, green line), while the bounded-state of compounds **4p** and **5c** displayed equilibration with obviously higher ﬂuctuations (2.6 Å and 2.73 Å for the complex of **4p**-DNA gyrase and **5c**-DNA gyrase, respectively) 3 red and orange lines. 

**Figure 5:**
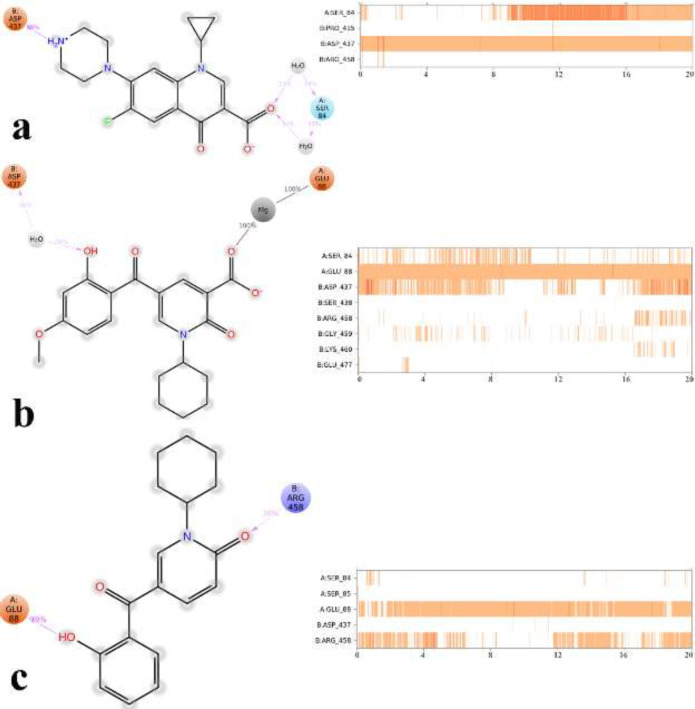
2D representation of ligand-residue interactions that occur during the 30% of simulation time which include DNA gyrase bound-state of ciprofloxacin (a), compound **4p** (b) and compound **5c** (c)

Based on the MD equilibrium state, ciprofloxacin stabilized at the DNA gate domain and formed H-bond with Ser84 and Asp437 through both the head carboxylate and the tail N atom of piperazine part of the molecule for about 34% and 84% of simulation time, respectively (Figure 5a). Figures 5b and 5c show compound **4p **and** 5c** tightly interacted with Glu88 by the carboxylic group and the 2-hydroxyl benzoyl moiety through Mg coordination and H-bond interaction, respectively for the significant amount of MD simulation time. In addition, like ciprofloxacin, Asp437 at GyrB subunit provided water-mediated H-bond with 2-hydroxy benzoyl moiety of compound **4p** for about 30% of MD simulation time, while in the case of compound **5c** Arg458 provided H-bond interaction with the 2-carboxy group of pyrimidone for about 38% of MD simulation time along. 

It is noteworthy that compounds **4p** and **5c** represent primary interaction as with fluoroquinolones with DNA gyrase through water-mediated and metal ion-bridge to Ser84, Glu88 from GyrA subunit and Arg458 and Asp437 located at GyrB subunit, which is the key residues in the DNA gate region of DNA gyrase. 

Ligand interacting with these residues at the GyrA and GyrB interface seems to be the most important for stabilizing the cleaved complex and may cause antibacterial activity through DNA gyrase inhibition (47, 48). Furthermore, Ser84 and Glu88 in *S. aureus* are the most commonly mutated in clinical isolates resistant to fluoroquinolones, which propose the reason why compounds provided lower activity over methicillin-resistant *S. aureus* (49).

*In-silico ADME properties of the most active synthesized compounds*

QikProp module of Schroedinger was used to calculate the essential pharmacokinetic properties including drug-likeness, metabolism, cell permeation, and bioavailability of the synthesized compounds.

Table 5 shows the “drug-likeness” of the synthesized compounds based on the “Lipinski rule of five”. It depicts there are no ROF violations and the synthesized compounds show a high probability of finding drug-like potential within these series. Furthermore, the absorption procedure, which affects the bioavailability of a compound, relies on the solubility and permeability of the compound (50). 

The computed parameter including the predicted aqueous solubility (log *S*_wat_), the predicted % human oral absorption (%HOA), and compliance to J*ϕ*rgensen’s famous “Rule of Three” (RO3) were measured (Table 6). According to Table 6, it was observed that all of the predicted descriptors are in acceptable value (51), which indicates the applied modification could emerge as a good candidate for drug discovery. 

**Table 1:** The** s**ynthesized 2-pyridone-3-carboxylic acids **4a-p**

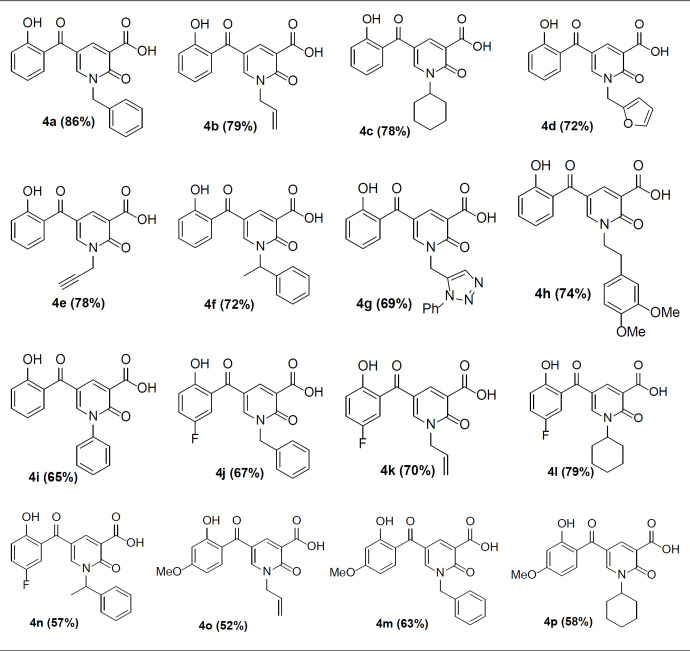

**Table 2:** Optimizing reaction condition for decarboxylation of 2-pyridone-3-carboxylic acids **4a**

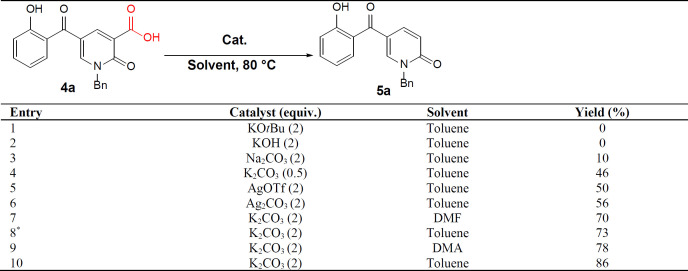

**Table 3:** The decarboxylated 2-pyridones **5a-o**

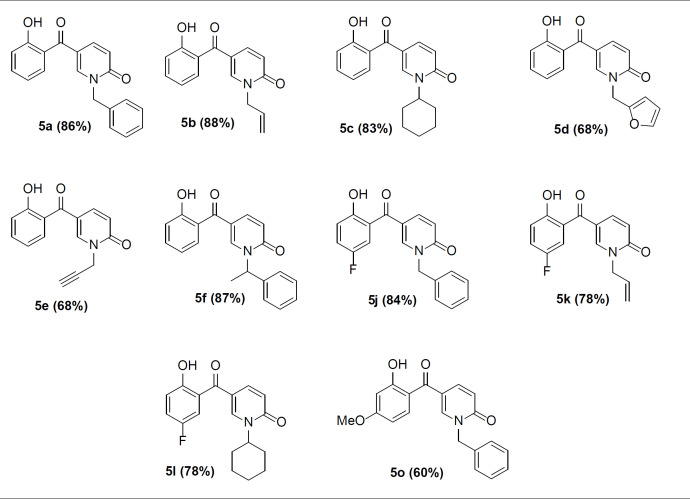

**Table 4:** *In-vitro* antibacterial activities of compounds **(4a-p**) and (**5a-o**).

**Compound**	**Minimum inhibitory concentration (MIC µg/mL)**						
	***E. coli***	***A. baumannii***		***S.aureus***	***MRSA***	***C. albicans***
**4a**	>500	500<	349.34		349.34	>500	
**4b**	>500	>500	4.67		>500	>500	
**4c**	341.36	>500	>500		>500	>500	
**4d**	>500	>500	>500		>500	>500	
**4e**	>500	>500	297.27		>500	>500	
**4f**	>500	>500	363.37		363.37	>500	
**4g**	>500	>500	416.39		>500	>500	
**4h**	12.79	>500	>500		>500	>500	
**4i**	>500	>500	>500		>500	>500	
**4j**	>500	>500	5.73		367.33	>500	
**4k**	>500	>500	>500		>500	>500	
**4l**	>500	>500	359.35		359.35	>500	
**4m**	>500	381.36	190.68		381.36	>500	
**4n**	>500	379.37	5.92		379.37	>500	
**4o**	329.31	329.32	10.29		82.33	>500	
**4p**	371.39	>500	2.96		>500	>500	
**5a**	305.33	>500	9.5		>500	>500	
**5b**	510.54	510.54	3.9		>500	>500	
**5c**	297.35	297.32	2.32		297.32	297.32	
**5d**	>500	295.29	73.82		>500	>500	
**5e**	>500	>500	>500		>500	>500	
**5f**	159.68	>500	>500		>500	>500	
**5j**	161.66	>500	161.66		>500	>500	
**5k**	>500	>500	>500		>500	>500	
**5l**	>500	>500	78.83		315.34	315.34	
**5o**	335.36	>500	167.68		>500	500>	
**Cefixime**	4	32	1		32	-	
**Ciprofloxacin**	0.5	64	0.25		64	-	
**Nystatin**	-	-	-		-	64	

*E. coli *PTCC 1399; *A. baumannii, clinical isolate*; *S. aureus *PTCC 1431; *MRSA*, clinical isolate; *C. albicans* ATCC 10231.

**Table 5:** The Lipinski rule of five properties of the most active compounds

**No.**	**Mw**	**HBD** ^a^	**HBA** ^b^	**Log** ***P*** ** o/w** ^c^	**rotor** ^d^	**ROF** ^e^
**4b**	299.282	0.000	5.750	2.016	6	0
**4j**	367.333	0.000	5.750	3.260	6	0
**4n**	379.368	0.000	6.500	3.116	7	0
**4o**	329.309	0.000	6.500	2.045	7	0
**4p**	371.389	0.000	6.500	2.806	5	0
**5a**	305.332	0.000	4.750	3.147	5	0
**5b**	255.273	0.000	4.750	2.127	5	0
**5c**	297.353	0.000	4.750	2.816	3	0
**ciprofloxacn**	331.346	1.000	6.000	0.280	1	0

^a^Number of average Hydrogen Bond Donor (recommended value 0.0 – 6.0).

^b^Number of average Hydrogen Bond Acceptor (recommended value 2.0 – 20.0).

^c^Predicted octanol/water partition coefficient (acceptable range from -2 to 6.5).

^d^Number of rotatable bonds (recommended value 2.0 – 20.0).

^e^Number of violations of Lipinski’s rule of five (maximum 4).

**Table 6:** The calculated ADME properties of synthesized compounds

**No.**	**Log** ***S***_wat_^a^	**HOA** ^b ^ **(%)**	**Metab** ^c^	**RO3** ^d^
**4b**	-2.695	71.4	2	0
**4j**	-4.210	73.1	2	0
**4n**	-3.919	72.5	3	0
**4o**	-2.870	62.1	3	0
**4p**	-4.285	67.8	2	0
**5a**	-3.720	94.6	3	0
**5b**	-2.372	88.7	3	0
**5c**	-3.883	95.6	2	0
**ciprofloxacin**	-3.793	75.7	0	1

^a^Predicted aqueous solubility in mol dm^−3^ (−6.5 – 0.5) (QPlogS > -5.7).

^b^Percentage human oral absorption (< 25% is poor and > 80% is high).

^c^Number of likely metabolic reactions (Primary Metabolites < 7).

^d^Number of violations of Jorgensen’s rule of three. Compounds with fewer (preferably no) violations of these rules are more likely to be orally available.

## Conclusion

New 2-pyridone-3-carboxylic acid deri-vatives were designed and synthesized through a three-component reaction between 3-formylchromone, primary amines, and Meldrum’s acid. Subsequently, a decarboxyl-ation reaction catalyzed by K_2_CO_3_ was performed in toluene, and all compounds were screened for their antimicrobial activity. IFD and MD study showed that the most active anti *S. aureus* are compounds **4p** and **5c** exhibiting primary interaction as with fluoroquinolones by cross-linking over DNA gyrase active site via metal ion bridge and H-bonding interaction with Ser84 and Glu88 from GyrA subunit along with Arg458 and Asp437 located at GyrB subunit. These interactions seem to be the most important ones for stabilizing the DNA cleaved complex and may cause antibacterial activity through DNA gyrase inhibition.
